# The hidden opportunity cost of time effect on intertemporal choice

**DOI:** 10.3389/fpsyg.2015.00311

**Published:** 2015-03-27

**Authors:** Cui-Xia Zhao, Cheng-Ming Jiang, Lei Zhou, Shu Li, Li-Lin Rao, Rui Zheng

**Affiliations:** ^1^School of Psychology, Shandong Normal UniversityJinan, China; ^2^College of Economics and Management, Zhejiang University of TechnologyHangzhou, China; ^3^Key Laboratory of Behavioral Science, Institute of Psychology, Chinese Academy of SciencesBeijing, China

**Keywords:** intertemporal choice, opportunity cost, time, SS choice, LL choice

## Abstract

An interesting phenomenon called “hidden opportunity cost of time effect” was detected in intertemporal choices. The majority of our participants preferred the smaller but sooner (SS) option to the larger but later (LL) option if opportunity cost was explicit. However, a higher proportion of participants preferred the LL to SS option if opportunity cost was hidden. This shift violates the invariance principle and opens a new way to encourage future-oriented behavior. By simply mentioning the “obvious” opportunity cost of alternatives, decision makers can be more informed in prioritizing their long-term goals rather than short-term goals.

## Introduction

Common examples of intertemporal choices in daily life are choosing between spending money on a vacation, investing in a superannuation fund for your retirement, and working on a promised paper now or later. In these cases, people have options that involve tradeoffs between costs and benefits occurring at different times. There is a general consensus in the literature on intertemporal choice that future outcomes are discounted (or undervalued) relative to immediate outcomes (Soman et al., 2005). Theoretical development in intertemporal choice has progressed steadily along a similar route as risky choice (Loewenstein and Prelec, 1992). Both lines of research have spawned a large amount of variant models, assuming a maximization principle; specifically, people calculate the mathematical expectation of each outcome and add them together before choosing the option that maximizes overall value or utility (Sun and Li, 2010; Rao and Li, 2011)

Opportunity cost is “the evaluation placed on the most highly valued of the rejected alternatives or opportunities” (Eatwell et al., 1998; Buchanan, 2008) or “the loss of other alternatives when one alternative is chosen” (Oxford English Dictionary, 2010) or “the value of the next-highest-valued alternative use of some resource (e.g., you spend time going to a movie, you cannot spend that time at home reading a book)” (Henderson, 2014). Fundamental to the discipline of economics is the issue of choice: choosing between scarce resources or alternatives (Meyer and Land, 2003). People, however, seem to ignore opportunity cost in the intertemporal choice involving money as the outcome (i.e., they ignore the fact that they could choose options detrimental to others). For example, Read et al. (2012) observed how the difference between a smaller but sooner (SS) option and a larger but later (LL) option is framed either as a total interest earned, as an interest rate, or as a total amount earned. They also examined whether the LL option is described as a consequence of the investment for SS option. These variations significantly modified the preferences of decision makers because they attracted the attention of decision makers to the opportunity cost. Previous researchers have found a hidden-zero effect in intertemporal choice, suggesting that explicitly referring to the hidden zero in each alternative (e.g., “Would you prefer [A] $5 today and $0 in 26 days OR [B] $0 today and $6.20 in 26 days?”) decreases the willingness of participants to choose impatient choices (i.e., “To receive $5 today”) (Magen et al., 2008). The explicit-zero format may draw attention to the opportunity cost of each choice, therefore encouraging people to choose the alternative that incurs a lower opportunity cost (i.e., to forgo the SS reward). Accordingly, we speculate that the explicitness of opportunity cost in each alternative can influence the choice of the decision maker.

Most previous studies on intertemporal choice use money as outcome (Read et al., 2005, 2012). However, opportunity costs are not restricted to monetary or financial costs. Lost time, real cost of output forgone, pleasure, or any other benefit that provides utility should also be considered as opportunity cost (Zauberman and Lynch, 2005; Frederick et al., 2009; Lynch et al., 2010; Spiller, 2011). Time is equally ubiquitous in the lives of people and is involved in intertemporal choice as outcomes. For instance, individuals choose between spending more time with family now and in the future. A student may also choose between spending 2 h on playing football with friends today and tomorrow. Although Benjamin Franklin encouraged equating time with money in his directive, “time is money,” research that compares time and money shows that people react to them differently (Zauberman and Lynch, 2005; DeVoe and Pfeffer, 2007, 2010, 2011; Mogilner, 2010; Aaker et al., 2011). For example, Zauberman and Lynch (2005) demonstrated that people expect slack (the perceived surplus of a given resource available to complete a focal task) for time to be greater in the future than in the present. Typically, this expectation of slack growth in the future is more pronounced for time than for money. According to the definition of opportunity cost, the opportunity cost of time and money is different in intertemporal choice. For money “Would you prefer [A] $5 today OR [B] $6.20 in 26 days?,” if you choose “[A] $5 today,” your opportunity cost is “0 in 26 days.” For time “Would you prefer [A] get 1 tour day tomorrow OR [B] get 2 tour days in a month?,” if you choose “[A] get 1 tour day tomorrow” your opportunity cost is “1 day studying time/working time” (because you spend time on tour, you cannot spend that time on studying or other things” and “0 day in a month.”

The above analysis leads us to better comprehend that the opportunity cost of time and money is different in intertemporal choice. That is, getting more money is clearly a reward because we lose nothing by gaining it. However, getting more free time is not clearly a reward because we lose the chance of spending that time on other activities. Therefore, when investigating the opportunity cost of time rather than money in intertemporal choice, we need to examine whether the time outcome is viewed as a gain/reward or a loss/cost.

Given that people's choices are heavily influenced by ways in which the alternatives are framed, even if the different frames are logically equivalent (Tversky and Kahneman, 1981), we hypothesize that representing each alternative in a different frame by explicitly referring to the hidden opportunity cost of time in each alternative will change the preference of participants. Specifically, the same time outcome which is seen as a gain/reward in one frame (hidden frame) can be seen as a loss/cost in another frame (explicit frame), for example, an extracurricular activity may be regarded as a gain if opportunity cost (e.g., the time spent on the extracurricular activity can be used on studying) is not explicated (hidden frame) but as a loss if opportunity cost is explicated (explicit frame), therefore leading to a mirror-image preference for “gain-seeking” vs. “loss-averse.” The subsequent experiments demonstrate our attempt to prove or disprove our hypothesis.

## Experiment 1

### Participants

A total of 106 undergraduate students majoring in psychology from Shandong Normal University participated in this study in a classroom setting (80 females, *M*_age_ = 20.31, *SD*_age_ = 0.98). All the participants provided oral consent and were given a small gift for their participation. All experiments were approved by the institutional review board of the Institute of Psychology, Chinese Academy of Sciences.

### Materials and procedure

A pair of intertemporal choices was prepared in Chinese and presented in questionnaire form in two versions.

The students were asked to imagine that they had recently studied intensively and that the teacher would like to offer an extracurricular activity to help them relax. They were given two options and asked to choose the one they preferred.

Version A: hidden frame—does not point out the opportunity cost of an extracurricular activity

A: participate in an extracurricular activity for 1 day tomorrow

B: participate in an extracurricular activity for 2 days in a week

Version B: explicit frame—points out the opportunity cost of an extracurricular activity

A': at the expense of 1 day of studying time, participate in an extracurricular activity for 1 day tomorrow

B': at the expense of 2 days of studying time, participate in an extracurricular activity for 2 days in a week

Participants were randomly assigned to either Version A or Version B. Version A had 56 participants, and Version B had 50 participants.

### Results and discussion

The results (Table 1) indicated that 4 of 56 participants preferred the SS option (Option A) in Version A, and 28 of 50 participants chose the SS option (Option A') in Version B. A 2 (hidden frame vs. explicit frame) × 2 (response) χ^2^ test revealed a significant relationship between version and preference: χ^2^_(1,96)_ = 29.91, *p* < 0.001, phi squared = 0.28. These results demonstrated that participants were more likely to choose the LL option if the opportunity cost of time was hidden but were more likely to choose the SS option if the opportunity cost of time was explicit. This result revealed that the proportion of participants opting for the SS option increased if the same problem was described in the explicit frame and suggested that a hidden opportunity cost of time effect was elicited.

**Table 1:** **Preference results as a function of problem frame in Experiment 1**.

**Problem frame**	**Preference results**	
	**SS option**	**LL option**
Hidden frame (*N* = 56)	4	52
Explicit frame (*N* = 50)	28	22

## Experiment 2

The preference shift in Experiment 1 violated descriptive invariance, which is one of the principles of normative decision making. Descriptive invariance claims that the different descriptions of an event or object should not change the preference of people. Tversky and Kahneman (1986) argued that the normative principles of decision making are generally satisfied when their application is transparent but are sometimes violated when not. The between-participants design used in Experiment 1 was viewed as a condition in which the decision making would not be transparent. Therefore, the intention of Experiment 2 was to examine whether the hidden opportunity cost of time effect was robust enough to survive using a within-participant rather than a between-participant design.

### Participants

A total of 70 undergraduate students (33 females, *M*_age_ = 20.39, *SD*_age_ = 0.95) from the School of Economics in Shandong Normal University participated in Experiment 2 in a classroom setting. All participants provided oral consent and were given a small gift for their participation.

### Materials and procedure

The same pair of intertemporal choices used in Experiment 1 was also used in Experiment 2 with minor modification. Specifically, the participants would not choose from the two options but would be asked to indicate their preference by circling a number on a seven-point scale ranging from 1 (definitely choose A/A') to 7 (definitely choose B/B').

Both versions of the choices were presented to each participant with other questions irrelevant to this study. The interval between the two tests was at least 7 days. The order of presentation of the two versions of choices was counter-balanced across the participants. Based on the within-participant design in Experiment 2, the application of decision making should be regarded as transparent.

### Results

Participants were more likely to choose the LL option (Option B/B') (*M* = 4.14, *SD* = 1.18) if options were presented in the hidden version than in the explicit version (*M* = 3.18, *SD* = 1.15), *F*_(1, 69)_ = 20.59, *p* < 0.001, η^2^ = 0.23. Therefore, Experiment 2 replicated the results of Experiment 1, although the decision making was transparent.

## Experiment 3

Based on the preference shift in Experiments 1 and 2, one might argue that “extracurricular activities” are actually seen as a reward and that the “hidden cost” is something in which, under the circumstances, students would have to be willing to risk offending their teacher and appear to be a slacker. Therefore, the extra cost of LL is NOT just an extra day of studying time—the real cost, as the situation is described, is the teacher's good opinion. Specifically, the teacher is offering one of two rewards, but in the explicit framing, the teacher is pointing out to the student that by accepting the LL offer, they will be slacking off and choosing to miss more studying time (“Here's your reward (LL)—but if you take it, you're a slacker”).

The aim of Experiment 3 is twofold: first, to help determine whether there is evidence to support the presumption that “extracurricular activities” is a reward; second, to exclude the potential confounding effect of the “hidden cost.” Accordingly, we modified the scenario that was used in Experiments 1 and 2, designed an athletic training scenario, and added a manipulation check following participants' response to each scenario.

### Participants

A total of 94 undergraduate students (83 females, *M*_age_ = 20.14, *SD*_age_ = 1.39) from the School of Psychology in Shandong Normal University participated in Experiment 3 in a classroom setting. All participants provided written consent and were given a small gift for their participation.

### Materials and procedure

Two hypothetical scenarios were prepared in Chinese (for Chinese versions see Appendix in Supplementary Material). One is the studying time scenario that was used in Experiments 1 and 2. We modified this scenario by replacing the “teacher” with “student representatives.” Specifically, the extracurricular activity would not be proposed by ***teacher*** but be proposed by ***student representatives***. To generalize the hidden opportunity cost of time effect on intertemporal choice, we designed a parallel training time scenario. In the training time scenario, the students were asked to imagine that they were athletes and they had recently trained intensively, and the ***teammates proposed*** to offer a tour to help them relax. Participants were asked to indicate their preference by circling a number on a seven-point scale.

Each participant was randomly assigned to answer either a hidden frame version or explicit frame version with each version having two scenarios: 47 participants responded to a hidden frame version, and the other 47 participants responded to an explicit frame version. Participants were asked to indicate their choice by circling a number on the 7-point scale ranging from 1 (definitely choose A) to 7 (definitely choose B). Then, participants were asked to rate to what extent they considered “1 day extracurricular activity, 2-day extracurricular activity, 1 day tour and 2-day tour” as loss or gain on a six-point scale (–3 for “large loss,” 3 for “large gain”). The two hypothetical scenarios and the corresponding manipulation check were presented as follows.

### Version A: hidden frame

#### Studying time scenario

The students were asked to imagine that they had recently studied intensively and that the ***student representatives*** propose to offer an extracurricular activity to help them relax. Participants were asked to indicate their preference by circling a number on a seven-point scale.

**Table:** 

Definitely choose A1						Definitely choose B1
1	2	3	4	5	6	7

*A1: participate in an extracurricular activity for 1 day tomorrow*. *B1: participate in an extracurricular activity for 2 days in a week*.

Do you consider “one/two-day extracurricular activity” as loss or gain? Please indicate your judgment by circling a number on a six-point scale.

**Table:** 

Large loss	middle loss	a little loss	a little gain	middle gain	large gain
−3	−2	−1	1	2	3

#### Training time scenario

The students were asked to imagine that they were an athlete and they had recently trained intensively, and the ***teammates proposed*** to offer a tour to help them relax. Participants were asked to indicate their preference by circling a number on a seven-point scale.

**Table:** 

Definitely choose A2						Definitely choose B2
1	2	3	4	5	6	7

*A2: participate in a tour for 1 day tomorrow*. *B2: participate in a tour for 2 days in a week*.

Do you consider “one/two-day tour activity” as loss or gain? Please indicate your judgment by circling a number on a six-point scale.

**Table:** 

Large loss	middle loss	a little loss	a little gain	middle gain	large gain
–3	−2	−1	1	2	3

### Version B: explicit frame

#### Studying time scenario

**Table:** 

Definitely choose A1'						Definitely choose B1'
1	2	3	4	5	6	7

*A1': at the expense of 1 day of studying time, participate in an extracurricular activity for 1 day tomorrow*. *B1': at the expense of 2 days of studying time, participate in an extracurricular activity for 2 days in a week*.

Do you consider “one/two-day extracurricular activity” as loss or gain? Please indicate your judgment by circling a number on a six-point scale.

**Table:** 

Large loss	middle loss	a little loss	a little gain	middle gain	large gain
−3	−2	−1	1	2	3

#### Training time scenario

**Table:** 

Definitely choose A2'						Definitely choose B2'
1	2	3	4	5	6	1

*A2': at the expense of 1 day of training time, participate in a tour for 1 day tomorrow*. *B2': at the expense of 2 days of training time, participate in a tour for 2 days in a week*.

Do you consider “one/two-day tour activity” as loss or gain? Please indicate your judgment by circling a number on a six-point scale.

**Table:** 

Large loss	middle loss	a little loss	a little gain	middle gain	large gain
−3	−2	−1	1	2	3

The experimental design was a 2 (hidden frame vs. explicit frame) × 2 (studying time scenario vs. training time scenario, nested within participants) between-subjects repeated factorial.

### Results

**Figure 1:**
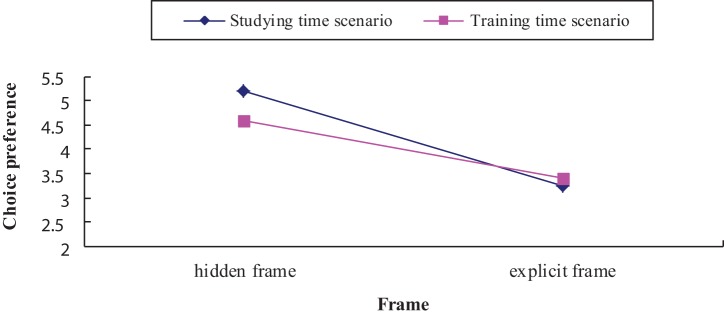
**Mean choice preference as a function of frame (explicit frame vs. hidden frame) and scenario (studying time vs. training time)**. Lower scores indicate preference for SS option; higher scores indicate preference for LL option; a score of 4 indicates no preference.

Mean choice preference (1 for definitely choose SS and 7 for definitely choose LL) as a function of frame (hidden frame vs. explicit frame) and scenario (studying time vs. training time) are shown in Figure 1. An ANOVA showed a main effect of the opportunity cost frame on the rated preference, with LL choices (2-day extracurricular activity/tour activity) being more likely to be chosen in hidden frame condition, whereas SS choices (1 day extracurricular activity/tour activity) were more likely to be chosen in an explicit frame [*F*_(1, 92)_ = 26.42, *p* < 0.001, η^2^ = 0.13]. Participants' preference for choice in the studying scenario was not different from that in the training scenario [*F*_(1, 92)_ = 0.58, *p* = 0.44, η^2^ = 0.003]. Moreover, we found no significant statistical interaction [*F*_(1, 92)_ = 1.56, *p* = 0.21, η^2^ = 0.008], suggesting that the “hidden opportunity cost of time effect” did not differ between the two parallel scenarios.

**Figure 2:**
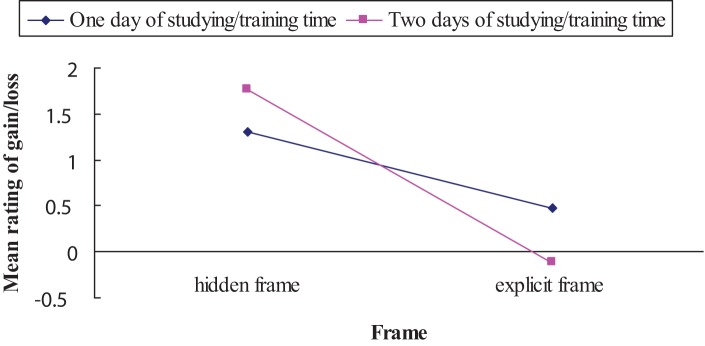
**Mean rating of gain/loss as a function of frame (explicit frame vs. hidden frame) and studying/training time (1 day vs. 2 days)**. Negative scores indicate that “studying/training time” was seen as *loss*; positive scores indicate that “studying/training time” was seen as *gain*.

Mean rating of gain/loss as a function of frame (hidden frame vs. explicit frame) and studying/training time (1 day vs. 2 days) are shown in Figure 2. An ANOVA showed a main effect of frame on mean rating of gain/loss, with scores of rating (*M*_hiddenframe_ = 1.53) being higher in hidden frame condition than those of rating (*M*_explicitframe_ = 0.36) in explicit frame condition [*F*_(1, 92)_ = 51.17, *p* < 0.001, η^2^ = 0.22]. The main effect of studying/training time (1 day vs. 2 days) was not significant [*F*_(1, 92)_ = 0.43, *p* = 0.51, η^2^ = 0.002]. Moreover, there was significant two-way interaction [*F*_(1, 92)_ = 30.16, *p* < 0.001, η^2^ = 0.14]. Further simple effect analysis revealed that in the hidden frame, the mean rating of 2 days of studying/training time (*M*_two days_ = 1.78) was significantly higher than 1 day of studying/training time (*M*_one day_ = 1.31) (*F* = 11.68, *p* = 0.001), whereas in the explicit frame, the mean rating of 1 day of studying/training time (*M*_one day_ = 0.48) was significantly higher than 2 days of studying/training time (*M*_two days_ = −0.12) (*F* = 18.92, *p* < 0.001). Considering that the negative scores indicate that “studying/training time” was seen as loss, the observed interaction suggests that the same time outcome in the LL option is more likely to be seen as a gain in the hidden frame, but it is more likely to be seen as a loss in the explicit frame. The results of the manipulation check, together with the results of choice preference, provide supportive evidence for our hypothesis that the same time outcome which is seen as a gain/reward in one frame (hidden frame) can be seen as a loss/cost in another frame (explicit frame), indicating a mirror-image preference for “gain-seeking” vs. “loss-averse.”

## General discussion

The present study investigated the opportunity cost of time rather than money in intertemporal choice. The difference between the time outcome and monetary outcome of intertemporal choice lies in that getting more money later is clearly a larger later reward, but getting more time is not necessary a larger later reward. We found that, unlike the monetary outcome, the same time outcome which is seen as a gain/reward in hidden frame can be seen as a loss/cost in explicit frame. The majority of our participants rejected the LL option which was seen as a loss/cost if opportunity cost of time was explicit. However, most participants preferred the LL option which was seen as a gain/reward if opportunity cost of time was hidden. We refer to this phenomenon as the hidden opportunity cost of time effect on intertemporal choice. This effect was detected regardless of whether its application was transparent, and the effect was highly consistent across all the settings tested in this paper. To our knowledge, the present research is the first to report this new phenomenon in intertemporal choice.

This research used time as the outcome and demonstrated that changing the construal of outcomes could affect the ability of individuals to consider the future consequences of their decisions. Although the presentation of the hidden and explicit opportunity costs of the time formats was logically equivalent, the different methods of presenting the options resulted in different results. The explicit opportunity cost of time formats resulted in high rates of SS choice and low rates of LL choice. This finding, together with other framing evidence reported previously in the literature (e.g., Levin, 1987; Levin and Gaeth, 1988; Lichtenstein et al., 1991; Li, 1998; Li and Xie, 2006; Li et al., 2007), contradict the principle of descriptive invariance (Tversky et al., 1988), which holds that individuals' decisions and preferences should not change solely because their options are described differently. The possible reason for this result is that the explicit opportunity cost format may draw attention to the opportunity cost of each choice; the “time” outcome is viewed as gain/reward in the hidden frame but loss/cost in the explicit frame, therefore encouraging people to choose the alternative that incurs a lower opportunity cost (i.e., the opportunity cost (loss) of 1 day of studying/training time was smaller than that of 2 days of studying/training time in explicit frame as shown in Figure 2). According to Magen et al. (2008), mentioning the “obvious” opportunity cost of alternatives may help decision makers choose in a more informed manner. They implied that opportunity cost changes the key decision input from the absolute value to the value of the option relative to the opportunity cost obtained. Additional research is required to elucidate the underlying mechanism of the effect observed in this study and to test this effect in real-world settings (e.g., time management). Moreover, given that opportunity cost is considered to be the evaluation placed on the most highly valued of the rejected alternatives or opportunities (Eatwell et al., 1998; Buchanan, 2008), further research is needed to investigate the plausibility and possible boundary conditions (e.g., long-term importance or weights placed on opportunity cost in each alternative) for the relationship between opportunity cost and SS/LL choice proposed here.

The actions of the grasshopper in Aesop's fable serve as an example of impulsiveness and short-sighted decision making (Milkman et al., 2008). People generally exhibit short-sighted preferences by choosing inferior, immediate rewards over ultimately superior but delayed future rewards (e.g., drug abuse, usurious loan, and extracurricular activities instead of studying). This short-sighted decision-making is implicated in many types of problematic behavior in healthy, normal, developing, and clinical populations, therefore leading to substantial individual and societal costs. Resisting impulsiveness and short-sighted tendencies to increase goal directedness often requires the exertion of control (e.g., Baumeister, 2002; Houben and Jansen, 2011; Tsukayama et al., 2012). The reported hidden opportunity cost of time effect, which is produced by simply changing wording, can help individuals conduct optimal long-term choices without the need for increased control.

### Conflict of interest statement

The authors declare that the research was conducted in the absence of any commercial or financial relationships that could be construed as a potential conflict of interest.
