# Supplementary material for: The hidden opportunity cost of time effect on intertemporal choice
Source: Front Psychol. 2015 Mar 27;6:311. doi: 10.3389/fpsyg.2015.00311 (PMC4376061; doi:10.3389/fpsyg.2015.00311)
Supplement: Supplementary file 1 [file DataSheet1.PDF]

Appendix

Version A: hidden frame

1. 最近同学们学习非常紧张，学生代表提议组织一次活动调节一下同学们紧张的学习生活，现有两种选择，请问你**更倾向**选择哪一个？请在最符合你真实想法的数字上打钩。

A.　明天得到 1 天的课外活动时间　　B.　一周后得到 2 天的课外活动时间

非常肯定选 A	比较肯定选 A	有点肯定选 A	不确定	有点肯定选 B	比较肯定选 B	非常肯定选 B
1	2	3	4	5	6	7

1.1 你觉得“**1 天的课外活动时间**”对你意味着损失还是获益？请在最符合你真实想法的数字上打钩。

很大损失	中等损失	有点损失	有点获益	中等获益	很大获益
-3	-2	-1	1	2	3

1.2 你觉得“**2 天的课外活动时间**”对你意味着损失还是获益？请在最符合你真实想法的数字上打钩。

很大损失	中等损失	有点损失	有点获益	中等获益	很大获益
-3	-2	-1	1	2	3

2. 想象你是一名运动员，最近训练非常紧张，有队友提议外出旅游调节一下紧张的生活，有两种选择，请问你**更倾向**于选择哪一个？请在最符合你真实想法的数字上打钩。

A　明天得到 1 天的旅游时间　　B　一周后得到 2 天的旅游时间

非常肯定选 A	比较肯定选 A	有点肯定选 A	不确定	有点肯定选 B	比较肯定选 B	非常肯定选 B
1	2	3	4	5	6	7

2.1 你觉得“**1 天的旅游时间**”对你意味着损失还是获益？请在最符合你真实想法的数字上打钩。

很大损失	中等损失	有点损失	有点获益	中等获益	很大获益
-3	-2	-1	1	2	3

2.2 你觉得“**2 天的旅游时间**”对你意味着损失还是获益？请在最符合你真实想法的数字上打钩。

很大损失	中等损失	有点损失	有点获益	中等获益	很大获益
-3	-2	-1	1	2	3

Version B: explicit frame

1. 最近同学们学习非常紧张，学生代表提议组织一次活动调节一下同学们紧张的学习生活，现有两种选择，请问你**更倾向**选择哪一个？请在最符合你真实想法的数字上打钩。

A. 明天牺牲 1 天的学习时间, 参加课外活动

B 一周后牺牲 2 天的学习时间，参加课外活动

非常肯定选 A	比较肯定选 A	有点肯定选 A	不确定	有点肯定选 B	比较肯定选 B	非常肯定选 B
1	2	3	4	5	6	7

1.1 你觉得"**1 天的课外活动时间**"对你意味着损失还是获益？请在最符合你真实想法的数字上打钩。

很大损失	中等损失	有点损失	有点获益	中等获益	很大获益
-3	-2	-1	1	2	3

1.2 你觉得"**2 天的课外活动时间**"对你意味着损失还是获益？请在最符合你真实想法的数字上打钩。

很大损失	中等损失	有点损失	有点获益	中等获益	很大获益
-3	-2	-1	1	2	3

2. 想象你是一名运动员，最近训练非常紧张，有队友提议外出旅游调节一下紧张的生活，有两种选择，请问你**更倾向**于选择哪一个？请在最符合你真实想法的数字上打钩。

A 明天牺牲 1 天的训练时间, 参加旅游。

B 一周后牺牲 2 天的训练时间，参加旅游。

非常肯定选 A	比较肯定选 A	有点肯定选 A	不确定	有点肯定选 B	比较肯定选 B	非常肯定选 B
1	2	3	4	5	6	7

2.1 你觉得"**1 天的旅游时间**"对你意味着损失还是获益？请在最符合你真实想法的数字上打钩。

很大损失	中等损失	有点损失	有点获益	中等获益	很大获益
-3	-2	-1	1	2	3

2.2 你觉得"**2 天的旅游时间**"对你意味着损失还是获益？请在最符合你真实想法的数字上打钩。

很大损失	中等损失	有点损失	有点获益	中等获益	很大获益
-3	-2	-1	1	2	3
